# Interactions between *Ascophyllum nodosum* Seaweeds Polyphenols and Native and Gelled Corn Starches

**DOI:** 10.3390/foods11081165

**Published:** 2022-04-18

**Authors:** Mauro Gisbert, Andrea Aleixandre, Jorge Sineiro, Cristina M. Rosell, Ramón Moreira

**Affiliations:** 1 Chemical Engineering Department, Universidade de Santiago de Compostela, Campus Vida, 15782 Santiago de Compostela, Spain; mauro.gisbert.verdu@usc.es (M.G.); jorge.sineiro@usc.es (J.S.); 2 Institute of Agrochemistry and Food Technology, Spanish Council for Science Research (CSIC), 46980 Valencia, Spain; andreaaleix91@gmail.com (A.A.); or cristina.rosell@umanitoba.ca (C.M.R.); 3 Department of Food and Human Nutritional Sciences, University of Manitoba, Winnipeg, MB R3T 2N2, Canada

**Keywords:** ABTS, adsorption, antioxidant activities, carbohydrates, DPPH, FRAP

## Abstract

The effect of several blending procedures between *Ascophyllum nodosum* seaweed flour (AF) and corn starch (CS) on the interactions between polyphenols and starch was studied in this paper. These methods comprised the blending of AF with native starch (NT) with previously gelled starch gel (GL) and promoting the gelling of corn starch in the presence of AF (CGL). Different AF–CS (g/g) ratios (from 1:0.5 to 1:25) were studied. The liquid phase was chemically characterized by polyphenols (TPC) and carbohydrates content. The antioxidant activity of the liquid phase after achieving the solid–liquid equilibrium was determined by DPPH, ABTS, and FRAP methods. The solid phase was characterized by FT-IR and SEM techniques. The Halsey model successfully fitted the equilibrium TPC in liquid and polyphenols adsorbed/retained by the solid phase of tested systems. NT samples showed lower polyphenols sorption than gelled samples. The differences found between samples obtained with GL and CGL methods suggested different interactions between polyphenols and starch. Specifically, physisorption is predominant in the case of the GL method, and molecular trapping of polyphenols in the starch gel structure is relevant for the CGL method. Results allowed us to determine the enhancement of the retention of polyphenols to achieve starchy foods with high bioactivity.

## 1. Introduction

The demand for gluten-free products is increasing since celiac disease affects around 1% of the world population. However, the food industry seems to be more focused on technological than nutritional quality [1,2]. A gluten-free diet can include starchy foods that usually show a high glycemic index and fat content together with low protein content [3]. FAO/WHO recommends reducing the intake of these high glycemic index meals since their continuous consumption increases metabolic disorders such as glucose intolerance, obesity, and type-II diabetes [4]. The design of new gluten-free products using natural and safe additives with health benefits to celiac patients is a current challenge [5].

The main polyphenols from seaweeds, named phlorotannins, are notorious antioxidant, anti-diabetic, and anti-hypertensive natural compounds [6]. They are being considered a valuable source of antioxidants by the drug and food industries [7]. Polyphenols extracted from *Ascophyllum nodosum* (*A. nodosum*) brown seaweed are potential food additives that may act as competitive inhibitors of digestive α–amylase and α–glucosidase enzymes, inhibiting the hydrolysis polysaccharides and thus reducing the glucose absorption levels [5]. Ingested phenolic compounds usually have low bioavailability due to their high sensitivity to gastrointestinal tract environments [6].

In vitro studies are easy, rapid, widespread, and reproducible methods to evaluate inhibitory phlorotannins capacities [8,9]. The inhibiting capacity of polyphenols against starch digestive enzymes (mainly α–amylase and α–glucosidase) is most often studied by mixing polyphenols with the enzyme, followed by the addition of substrates promoting polyphenols–enzymes interactions. In real food consumption, the inhibition of starch digestive enzymes can be achieved either by taking digestive enzymes inhibitor pills of chemical–handmade (i.e., acarbose, miglitol, and voglibose) or by increasing the intake of natural polyphenols [4]. An alternative strategy may be the integration of polyphenols within food matrices; therefore, it is necessary to ensure the polyphenol contact with digestive enzymes in the small intestine. The polyphenols–starch interactions might influence their enzymatic inhibitory capacities, stressing the relevance of the selection of the most adequate methods to ensure optimal intestine delivery, bioavailability, and beneficial health of this new generation of foods [10].

Phlorotannins are easily affected by oxygen, pH, ions, light, and temperature [11]. As reported by Guo et al. [12], the design and production of foods with added phlorotannins have synergic beneficial effects since meals quality is improved and the polyphenols deterioration rate is reduced [12]. Starch matrices have been demonstrated to act as shelters for various food bioactive compounds such as fatty acids, phenolic compounds, vitamins, and probiotics [13]. Polyphenols–starch blends have been largely studied because starches are low cost, abundant, edible, non-allergenic, and classified as Generally Recognized As Safe (GRAS), film-forming capacities, emulsification properties [14]. Nowadays, starchy-based encapsulation is being applied for diverse biomedical and industrial applications [6]. The polyphenols can interact with starch and other macromolecules in reversible (solubilization, emulsification, film-forming, and sorption) or irreversible ways (crosslinking, acetylation, esterification, and oxidation) [12].

These interactions are affected not only by the specific polyphenol structure but also by the different experimental conditions and/or the nature of the polymer [15]. Starch and polyphenols complexes modified starch characteristic structure, rheological, thermal, and solubility properties [16]. Therefore, these modifications influence the retention and release of these antioxidant components. According to Aleixandre and Rosell [17], by adding phenolics during corn starch gelatinization, its retention could vary depending on the polyphenol characteristics, as well as on starch properties.

There is extensive literature reporting mechanisms of sorption, complexation, interaction, or encapsulation of polyphenols on several starchy materials (wheat, corn, potato, yuca, green pea, beans), using a wide variety of bioactive sources (tea leaf, yerba mate, sorghum) and using different immobilization methodologies [6,10]. However, studies considering polyphenols from seaweeds in these new bioactive starchy products are scarce. It is proposed to add the seaweeds to recipes, acting as a two-step process with an initial solid–liquid extraction of polyphenols from *A. nodosum* seaweeds powder (AF) followed by the sorption/complexation process between polyphenols and starch.

The use of whole raw material as a bioactive compounds source involves a reduction in time, costs, and energy consumption of processing since extraction and purification processes are omitted [18]. Conversely, seaweeds can contain undesirable components, such can be an eventual excessive iodine content, which oral intake must be limited, or some heavy metals, depending on their origin. Additionally, the special taste of brown seaweeds is a critical aspect of the production and consumer acceptance of these seaweed-derived products. A proper selection of the seaweed raw material (species, origin, seasonal harvesting), processing (drying, preservation), and extraction and purification of bioactive compounds, together with their integration in the food matrices, can be used to diminish these deficiencies [5]. Moreover, seaweed–starch complexations have been demonstrated to extend food shelf-life, protect bioactive molecules against gastrointestinal tract conditions, and release them into the intestine [14]. The characteristics of the starch gelatinization process in the presence of seaweeds (or polyphenols from them) are proposed as key aspects of the bioactive immobilization of starchy materials in the present paper. Increasing the knowledge of this process would suppose a relevant advance in food science and technology since products with tunable sensory, bioactivity, and rheological properties could be achieved with simple operational and manufacturing control [19].

*Ascophyllum nodosum* seaweed flour (AF) bioactive molecules and corn starch (CS) interactions were studied in this paper, employing three different methods (noted as NT, GL, and CGL). The NT method consisted of blending AF and native CS. The GL method consisted of blending AF and pregelatinized CS. Finally, in the CGL procedure, CS was gelatinized in the presence of AF. Different proportions of AF and CS were proposed (1:25, 1:2, 1:1, and 1:0.5), and their aqueous phases were chemically characterized by bioactive compound content (polyphenols and carbohydrates) and by antioxidant activities (DPPH, ABTS, and FRAP). Solid was characterized by FT-IR and SEM techniques.

## 2. Materials and Methods

### 2.1. Chemicals

All reagents used for characterization were analytical grade. Sulfuric acid, phenol, and iron sulfate were supplied by Merck (Darmstadt, Germany). 2,2-diphenyl-1-picrylhydrazyl (DPPH), 2,4,6-*tris* (2-pyridyl)-*S*-triazine (TPTZ), potassium hydroxide, potassium chloride, iron (III) chloride, sodium chloride, potassium dihydrogen phosphate, sodium phosphate, sodium azide, phloroglucinol, and sodium acetate were from Millipore Sigma (St. Louis, MO, USA). 2,2-azinobis (3-ethylbenzothiazoline-6-sulfonic acid) diammonium salt (ABTS), sodium carbonate, Folin–Ciocalteau reagent, chlorohydric acid, glucose, Trolox, sodium hydroxide, and methanol were from Panreac (Barcelona, Spain).

### 2.2. Raw Material

Food-grade native corn starch (Tate and Lyle PLC, London, UK) of 95% purity (20.3% amylose content), 8.1% ± 0.2% (d.b) moisture content was used. Fresh *Ascophyllum nodosum* seaweed (*A. nodosum*) from Galicia’s coasts (NW of Spain) was harvested in November 2019, supplied by Mar de Ardora S.L. company (Ortigueira, Spain), dried in a hot air convective dryer (Challenge 250, Angelantoni, Massa Martana, Italy) at 50 °C, with a constant relative humidity of 30% and air velocity at 2 m/s. Dried *A. nodosum* was ground in an ultra-centrifugal mill (ZM200, Retsch GmbH, Haan, Germany). *A. nodosum* seaweed powder (AF) was stored at 4 °C with a final moisture content of 10.0% ± 0.1% (d.b) until its use.

### 2.3. Seaweeds–Starch Interaction Methods

**Figure 1:**
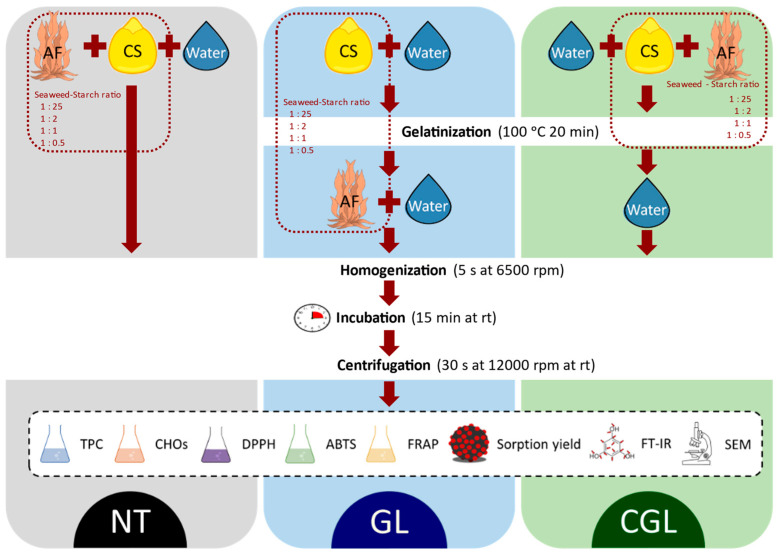
Process scheme summarizing methodology. NT method: AF (seaweeds flour) and native corn starch (CS) blending; GL method: AF and gelled CS blending; CGL method: CS gelatinized in the presence of AF.

Three methods (NT, GL, CGL), Figure 1, for CS and AF blending were tested with slight modifications with respect to that proposed by Wang et al. [10]. The NT and GL methods were assayed to study the effect of CS structural features on the interactions with bioactive compounds from AF. Conversely, the CGL method was based on two stages: first, bioactive compounds extraction from AF together with their interactions with CS during the gelatinization step; second, partial leaching of bioactive molecules from the gel after water addition. The objective of this last method was to simulate the behavior of bioactive compounds during the heating steps during starchy products processing. So, NT consisted of the native CS and AF blending; gelled CS was blended with AF in GL, and CS was gelatinized in the presence of AF in the CGL method. AF and CS control samples were also analyzed to determine the chemical characteristics of both powders.

Starch gelatinization was performed according to the method reported by Wang et al. [20]. Aqueous CS (20% w/w) was immersed in a boiling water bath at 100 °C for 20 min. The CGL gelatinization was carried out under the same conditions for AF and CS blends. After the gelatinization stage, the samples were cooled (20 min) at room temperature (rt, 20 ± 1 °C) until gel temperature was lower than 35 °C. Subsequently, GL and CGL samples were homogenized using a homogenizer (IKA-Werke, Staufen, Germany) with 3 pulses of 5 s at 6500 rpm. Broken gel was blended with seaweeds and additional water in the GL method, whereas only distilled water was added to obtain CGL samples.

The liquid-solid ratio was set at 100 g_W_/g_AF_ (g of water/g of seaweed). AF and CS content varied to obtain different *A. nodosum* flour-to-cornstarch blending ratios (1:25, 1:2, 1:1, and 1:0.5) corresponding to 4, 50, 100, and 200 g_W_/g_CS_, (g of water/g of starch), respectively. A ratio of 1:25 was used as the average proportion studied by bakery products [1]; meanwhile, the remaining ratios were studied to determine more adequately the molecular interactions. These AF–CS mixtures in water were homogenized and rested for 15 min at 20 °C (Figure 1). The elapsed time necessary to achieve a solid/liquid pseudo-equilibrium (between water and seaweed flour) was previously determined and was shorter than 15 min. This procedure agrees with the kinetics determined between potato maize and procyanidins by Qiu et al. [6]. The phytochemicals content was analyzed after samples centrifugation at 12,000 rpm for 30 s at rt, followed by filtration of the supernatant through a 0.45 µm microfiber filter (Merck, Darmstadt, Germany).

### 2.4. Chemical Characterization

Chemical characterizations were carried out with a spectrophotometer (Genesis 10S UV, Thermo Fisher Scientific, Waltham, MA, USA) at least in triplicate. Total polyphenol content (TPC) was determined using phloroglucinol as standard following the method proposed by Singleton and Rossi [21] based on the Folin–Ciocalteau reagent reaction with hydroxyl groups, measured spectrophotometrically at 765 nm. The TPC values were given as g of phloroglucinol equivalents per liter (g_PE_/L). TPC values provided by AF in the AF–CS blends were calculated using Equation (1):

(1) $$\mathrm{TPC}={\mathrm{TPC}}_{\mathrm{AF}-\mathrm{CS}}-{\mathrm{TPC}}_{\mathrm{CS}}$$

where TPC_AF–CS_ is the polyphenols content measured in the liquid phase of AF–CS blends and TPC_CS_ is the corresponding polyphenols content of CS control samples.

The total content of carbohydrates (CHOs) was spectrophotometrically determined at 485 nm, applying the method reported by Dubois et al. [22], using glucose as standard. CHOs results were given as g of glucose equivalents per liter (g_GE_/L). The antioxidant activity of the aqueous phases was determined using DPPH, FRAP, and ABTS methods, expressing the results as the equivalent activity of the Trolox standard in micromole units (µM_TE_). DPPH scavenging activity determinations were performed following the methodology proposed by Brand–Williams et al. [23]. The scavenging activity of samples was determined by reduction in the absorbance at 515 nm after 30 min of incubation at 20 °C. The ABTS method was carried out following the Re et al. [24] method, measuring absorbance at 734 nm after 15 min of incubation at 20 °C. The iron cation reduction capacity (FRAP) of the extracts was performed according to the Benzie and Strain [25] procedure, measuring absorbance at 593 nm after 30 min of incubation at 20 °C.

### 2.5. Bioactive Compounds Adsorption

Equilibrium sorption yield, Y_P_ (%), of polyphenols was evaluated by Equation (2), and the polyphenols adsorbed by CS, q (mg_PE_/g_CS_), were determined by Equation (3):

(2) $$Y_{P}=\left(1-\left(\frac{\mathrm{TPC}}{{\mathrm{TPC}}_{\mathrm{AF}}}\right)\right)\cdot 100$$

(3) $$q=\left({\mathrm{TPC}}_{\mathrm{AF}}-\mathrm{TPC}\right)\;\left(\frac{V}{m_{\mathrm{CS}}}\right)$$

where TPC_AF_ is the polyphenol content (g_PE_/L) of the aqueous phase corresponding to AF control samples, V is the liquid volume (L), and m_CS_ is the final CS mass (g), evaluated by means of Equation (4):

(4) $$m_{\mathrm{CS}}={\;m}_{\mathrm{CSi}}-\left({\mathrm{TPC}}_{\mathrm{CS}}-\;\mathrm{CHOs}\right)\;V$$

where m_CSi_ is the initial CS mass (g) and CHOs the carbohydrate content (g_GE_/L) of the liquid phase released from starch (adsorbent).

### 2.6. Fourier Transform Infrared Spectrophotometry (FT-IR)

AF–CS samples at the intermediate ratio (1:1) were analyzed by FT-IR to characterize the AF–CS interactions. FT-IR spectra were recorded with a Bruker FT–MIR model Vertex 70 V spectrometer. The wave number range was set in the range of 4000 to 50 cm^–1^. Samples were blended with KBr and compressed into disks. FT-IR spectra treatment was carried out with Omnic 7.1 software (Thermo Scientific, Waltham, MA, USA).

### 2.7. Scanning Electron Microscopy (SEM)

AF–CS (1:1 ratio) samples were freeze-dried for 36 h (−55 °C and 50 Pa), and their microstructure was analyzed by scanning electron microscopy (SEM). Samples were sputtered with iridium using a vacuum metallizer/shader model Q150T S (Quorum Technologies Ltd., Lewes, UK) with a thickness layer of 5–10 nm was deposited. Samples micrographs were obtained using a scanning electron microscope (FESEM Ultra Plus with EDX, Zeiss, Jenna, Germany) at 3 Kv using a SE/InLens secondary electron detector.

### 2.8. Statistical Analysis

Statistical analysis was carried out by IBM SPSS statistics 27 (SPSS Inc., Chicago, IL, USA) software. A one-way analysis of variance (ANOVA) was assessed based on a confidence interval of 95% (*p* < 0.05) using a Duncan test. The experimental results were treated and plotted on Microsoft Excel (Microsoft Corporation, Redmond, WA, USA). All experimental results were expressed as mean ± standard deviation of triplicate experiments (*n* = 3).

## 3. Results

**Figure 2:**
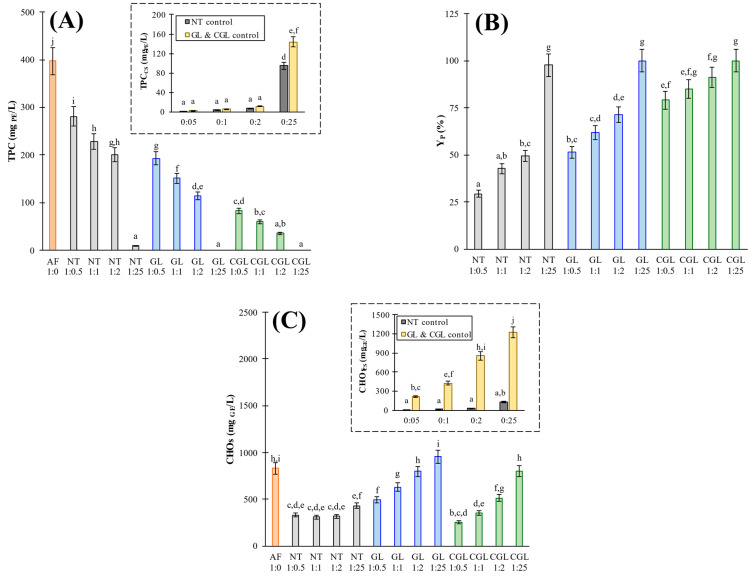
TPC values (**A**), sorption yields, Y_P_ (**B**), and CHOs values (**C**) of AF (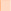), CS (native (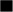), and gelled (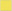)) control samples and AF–CS blending methods of NT (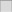), GL (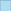), and CGL (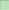) samples. NT method: AF (seaweeds flour) and native corn starch (CS) blending; GL method: AF and gelled CS blending; CGL method: CS gelatinized in the presence of AF. Different letters indicate significant (*p* < 0.05) differences among samples.

The aqueous phase from *Ascophyllum nodosum* seaweed powder (AF), corn starch (CS), and AF–CS blends were chemically characterized, determining polyphenols content (TPC), carbohydrates content (CHOs), and their antioxidant activities by DPPH, ABTS, and FRAP methods. TPC values (A), sorption yields, Y_P_ (B), and CHOs values (C) of AF (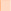), CS (native (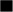), and gelled (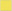)) control samples and AF–CS blending methods of NT (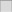), GL (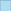), and CGL (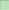) samples are summarized in Figure 2 where different letters indicate significant (*p* < 0.05) differences among samples. For the CS control, it was necessary to evaluate according to CS structures, consequently, native (NT samples) and gelled CS (GL and CGL samples) controls were employed in each case.

### 3.1. Aqueous Phase Chemical Characterization

#### 3.1.1. Total Polyphenolic Content (TPC)

The average total polyphenols content of the *A. nodosum* control sample in solution (TPC_AF_) was 396.9 ± 28.6 mg_PE_/L. The TPC evaluation of corn starch control samples was performed to correct the values of total polyphenols for some reducing power that starch-derived carbohydrates might have on the Folin–Ciocalteau reagent. Starch concentration almost did not affect the TPC values obtained (Figure 2A, ratios 0:0.5, 0:1, and 0:2), except the value at low liquid-to-solid ratios (blend 0:25), which was significantly (*p* < 0.05) higher. Slight differences were found between TPC from native CS (from 1.9 ± 0.1 to 94.9 ± 6.8 (mg_PE_/L) and gelled CS (from 2.9 ± 0.2 to 143.4 ± 10.3). The control samples evaluation showed that starch, either gelled or not, did not significantly contribute to final TPC values, but it was not negligible for 0:25 samples. The obtained TPC values from both control samples separately (AF and CS) rendered higher values than those measured with the corresponding blends, Figure 2A. TPC values of the NT blends ranged from 9.4 ± 0.7 to 280.5 ± 20.2 mg_PE_/L, GL samples showed lower content, from 0.02 ± 0.01 to 192.9 ± 13.9 mg_PE_/L, and CGL showed the lowest values (at constant AF–CS ratio) with a maximum value of 82.8 ± 6.0 for 0:0.5 ratio (Figure 2A). These results indicate that the polyphenols released from seaweed to the liquid phase interacted with CS (native or gel) structures remaining bound to or trapped in the solid phase. For the three assayed methods (NT, GL, and CGL), the higher the corn starch ratio, the lower the net TPC values in liquid; thus, negligible values were obtained at 1:25 ratio samples with starch gels. GL values corresponding to gelled CS showed lower (*p* < 0.05) polyphenols content in relation to NT. The different available sorption surface areas between native starch (granular structure) and gel starch could explain these differences [10].

The blending procedure that involves the gelling of starch in the presence of seaweeds polyphenols (CGL) caused a dramatic reduction in TPC values. The NT and GL methods involved the direct contact between AF and CS (native or gelled) at rt for 15 min; meanwhile, CGL put them in contact during the gelatinization process carried out at 100 °C (20 min), a cooling step (20 min), and the final dispersion and homogenization in water. During CS gelling with the presence of AF, both the *A. nodosum* particles and extracted polyphenols can be trapped within the gel structure during gels formation and subsequent retrogradation [19]. Another factor that could be responsible for the decrease in polyphenols content is the thermal treatment, which could promote the polyphenols partial decomposition [26,27] or the formation of new bonds and substances [28].

Wang et al. [10] studied native starch, starch gel, and gelatinized corn starch in the presence of tannic acids, reporting that the formation of bindings between tannins and gelled starch was more abundant than between tannins and native starch. The gelling of starch in the presence of tannic acids, such as “CGL blend” here, also showed notorious lower TPC values. Notoriously higher retention of polyphenols in gelled starch (GL and CGL), as compared with native starch, was also reported by Barros et al. [28]. In fact, these authors obtained different bioactive compounds content for oven-dried (at 105 °C) and for freeze-dried starchy–sorghum matrices. Initial TPC is a critical aspect in equilibrium adsorption experiments; in the current study, this value (396.9 ± 39.7 mg_PE_/L) was lower than those employed by Wang et al. [10] (10, 20, and 30 mg of tannic acids per mL) and are comparable to those employed by Barros et al. [28], with initial 485 mg of gallic acid equivalents per g of sorghum. Nevertheless, both authors obtained similar trends. Even working with higher liquid-to-solid ratios, the polyphenols extraction was enough to show relevant and measurable interaction with native and gelled corn starch.

TPC from the aqueous phase of AF–CS blends and AF and CS control samples were used to determine the sorption yield (Y_P_) of the samples (Figure 2B). Y_P_ varied between 29.3 ± 1.8 (NT 1:0.5) and 99.8 ± 5.9 (both GL and CGL 1:25). Y_P_ values showed higher retention capacities of the gelled corn starch (GL and CGL), with similar trends for the three methods tested, where the higher the CS content, the higher the polyphenols retention yield. NT samples showed lower sorption yield values to the lower active surface area of native starch to interact with polyphenols [29]. To understand both effects, AF–CS blending methods (NT, GL, and CGL) and CS structure (native and gelled) on polyphenol sorption, different ratios of Y_P_ (GL/NT, CGL/NT, and CGL/GL) were calculated, and the corresponding values are presented in Table 1.

The lowest ratios value was 1 at ratio 1:25 because the adsorption yield was near 100% when the highest starch amount was employed. The highest values were 1.75 ± 0.07, 2.70 ± 0.05, and 1.54 ± 0.08 for GL/NT, CGL/NT, and CGL/GL ratios, respectively, when the lowest adsorbent (1:0.5 ratio) was used. These values indicated that the use of starch gel increased almost twice the polyphenols retained in comparison to native starch. These values could be related to the higher surface area developed in the gel structure in comparison to the granular structure. When the formation of starch gel took place in the presence of seaweeds, flour increased by almost 3 and 1.54 times regarding the NT method and GL methods, respectively. This notorious increase could be explained by additional retention of polyphenols beyond adsorption, such as entrapment or encapsulation of bioactive molecules in the gel structure. As the amount of starch increased (1:1 and 1:2 ratios), the differences decreased due to the respective increase in sorption yields.

**Figure 3:**
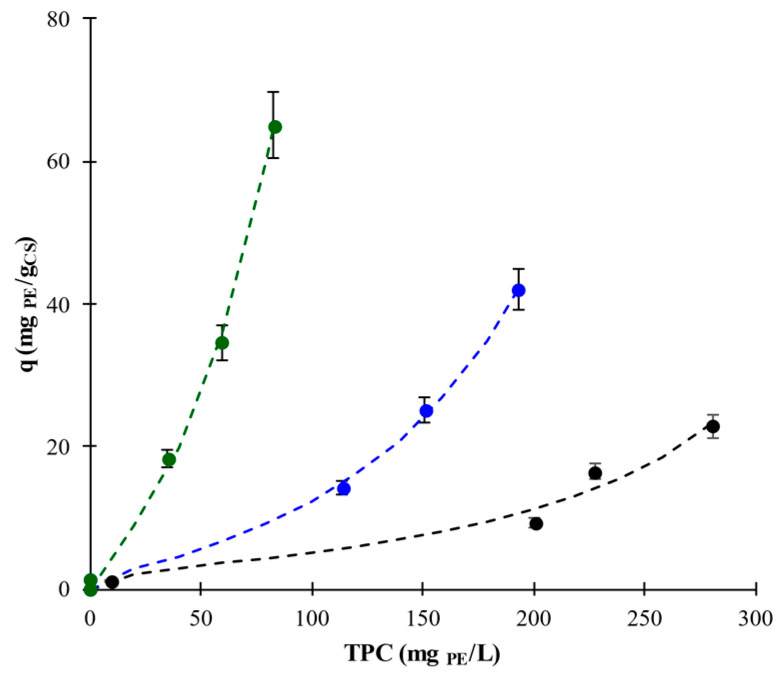
Adsorbed/retained polyphenols by CS, q, vs. TPC of aqueous phase for AF–CS samples (NT, 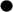, GL, 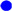, and CGL, 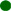) at 20 °C. Lines are the Halsey model (Equation (5)).

Polyphenols retained by CS, q (m_gPE_/g_CS_), determined by means of Equation (3) against TPC values of the liquid phase, are plotted in Figure 3. For NT, GL, and CGL samples, q values were lower than 22.9 ± 1.6, 42.0 ± 2.9, and 65.0 ± 4.5, respectively, and were achieved when the lowest starch amount was employed. Furthermore, q values were near zero, employing a 1:25 ratio because yields were around 100%. Figure 3 clearly shows the enhancement of the polyphenol–starch affinity in GL and CGL samples regarding NT samples. In example, at q = 15 mg_PE_/g_CS_, equilibrium is achieved at TPC of 240, 115, and 35 mg_PE_/L of TPC for NT, GL, and CGL samples. These results confirmed that low sorption surface area was available when native corn starch granules were employed in comparison to the area developed by starch gel structures in GL and CGL samples. In all cases, the shapes, convex to the abscissa axis over the tested range, of equilibrium adsorption/retention isotherms of polyphenols on starch allowed their classification as III according to the BET classification [30]. An empirical two-parameters model, the Halsey model, Equation (5), was employed to fit the experimental data [31].

(5) $$q\;={\left(\frac{-A}{\ln\;\left(\frac{\mathrm{TPC}}{{\mathrm{TPC}}_{\mathrm{AF}}}\right)}\right)}^{\frac{1}{B}}$$

where A and B are the fitting parameters.

Table 2 shows the A and B values of Equation (5) for NT, GL, and CGL samples. According to the values of coefficient of determination (*R^2^* > 0.98) and root mean square error (E_RMS_ < 3.6), the goodness of fitting can be considered acceptable; Figure 3 shows the modeled values. Values of parameter A varied in a narrow range (from 5.2 ± 0.7 to 6.1 ± 0.4) with the blending procedure, but exponent B^−1^ varied from almost linear (1.1) for NT, quadratic (1.9) for GL, and cubic (3.0) for CGL.

#### 3.1.2. Total Carbohydrate Content (CHOs)

CHOs (mg_GE_/L) values (Figure 2C) were notoriously high for gelled corn starches and provided by both raw materials, *A. nodosum* seaweed flour and corn starch. CHOs increased with CS content, indicating that CS carbohydrates were partially solubilized, mainly because of the thermal treatment, as can be deduced from the results of control gelled samples (GL and CGL controls in Figure 2C). CHOs evaluation was made mandatory to handle data comparing AF–CS blends with control samples of AF and CS. The AF control sample (1:0) showed an initial CHOs value of 830.4 ± 59.8 mg_GE_/L while NT control values varied between 8.2 ± 0.6 and 129.5 ± 9.3; meanwhile, gelled CS methods (GL and CGL) ranged from 213.2 ± 15.4 to 1223 ± 88 for 1:0.5 to 1:25 AF–CS ratios, respectively. CHOs significant (*p* < 0.05) differences stressed the notorious effect that gelatinization had on CHOs signals.

At constant AF–CS ratios, CHOs values were significantly (*p* < 0.05) higher in GL (495.1 ± 35.6 to 955.1 ± 68.8) in comparison to CGL (252.0 ± 18.2 and 804.6 ± 57.9) and samples (332.7 ± 24.0 to 431.9 ± 31.1). Higher CHOs values of GL samples were associated with the leaching of some polysaccharides (mainly amylose) during starch gelatinization and, in addition, with the extracted carbohydrates from AF, increased the total carbohydrates content; this effect was clearly evidenced in assays performed at 1:25 ratio. Low CHOs values determined in CGL samples can be again explained by the gel trapping effect. The formation of CS gel in the presence of AF limited the release of carbohydrates from algae to the liquid phase, which notoriously (*p* < 0.05) reduced CHOs in AF–CS samples. Moreover, thermal deterioration during gelatinization could also partially alter sugars [28], making them not detectable. Scarce sugar content evaluation is carried out in the literature; however, it is necessary to correctly evaluate the adsorbent amount at equilibrium and is also useful to understand the physical and chemical phenomena involved during the formulation of these new bioactive starchy foods since many of *A. nodosum* carbohydrates have demonstrated relevant phytochemical features.

#### 3.1.3. Antioxidant Activities (DPPH, ABTS, and FRAP)

**Figure 4:**
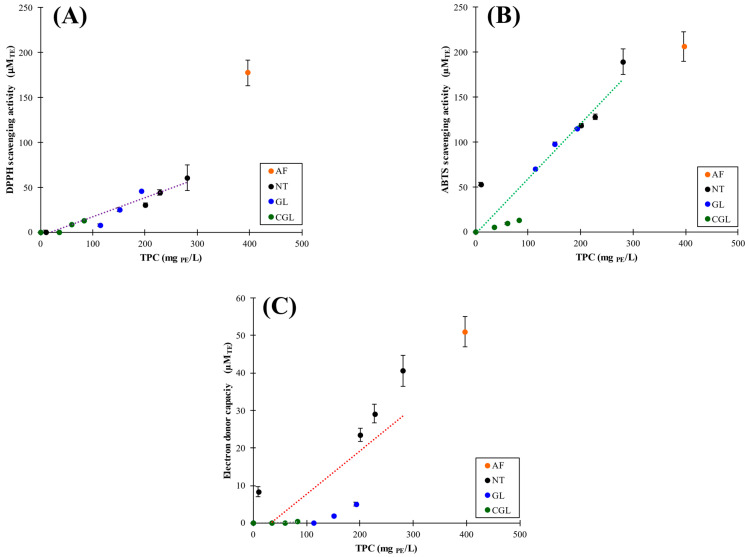
Antioxidant activities determined by DPPH (**A**), ABTS (**B**), and FRAP (**C**) vs. TPC in control (AF (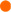)) and AF–CS samples (NT, 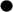, GL, 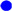, and CGL, 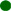 methods).

Antioxidant activities were determined by DPPH (Figure 4A) and ABTS (Figure 4B) radical scavenging activities and FRAP as a method for electron donor capacity (Figure 4C) of aqueous phases of tested samples at equilibrium. As expected, the highest antioxidant activity was obtained for the AF control sample (Figure 4A–C, isolated dots). The lowest antioxidant activity values were obtained for CGL samples. This last result could be related to the thermal deterioration of polyphenols during heating treatment and the entrapment of bioactive compounds into the starch gel.

An acceptable linear correlation (*R*^2^ = 0.93) between DPPH and TPC values, without distinctions among employed blending procedure, was found, indicating that scavenging activities of polyphenols measured by this method depend only on TPC, and the possible deterioration of bioactive compounds is not detected. Nevertheless, to achieve suitable linear correlations (*R*^2^ > 0.91) for ABTS-TPC results, experimental data had to be separated into two sets according to AF thermal treatment, subjected (CGL) and non-subjected (NT and GL) to the heating process, and consequently employing this more sensitive method in different antioxidant activities trends with TPC could be successfully established. Finally, a linear correlation for FRAP-TPC data was only found for NT samples (*R*^2^ = 0.93) because FRAP values corresponding to GL and, particularly, CGL methods were very low. This last result demonstrated that the most bioactive polyphenols became notoriously trapped by CS gel, remaining with low bioactivity in the liquid phase. Other researchers also reported linear correlations in polyphenol–starch blends [32]. In this manner, AF–CS matrices can be considered promising polyphenols shelters against the digestive tract [12,13]. Moreover, the different CGL-TPC trends (for ABTS and FRAP methods) with very low antioxidant activities could also be related to partial thermal degradation (oxidation, polymerization, etc.) of polyphenols during processing that significantly reduces the bioactivity of non-adhered phytochemicals on the starch surface.

### 3.2. Fourier Transform Infrared Spectrophotometry (FT-IR)

The FT-IR technique was carried out to evaluate the nature of bindings formed between CS and polyphenols by means of the analysis of AF, CS, and AF–CS blends (1:1 ratio) from NT, GL, and CGL methods. FT-IR can provide relevant structural information and was previously employed in polyphenols–starch systems [10,33].

**Figure 5:**
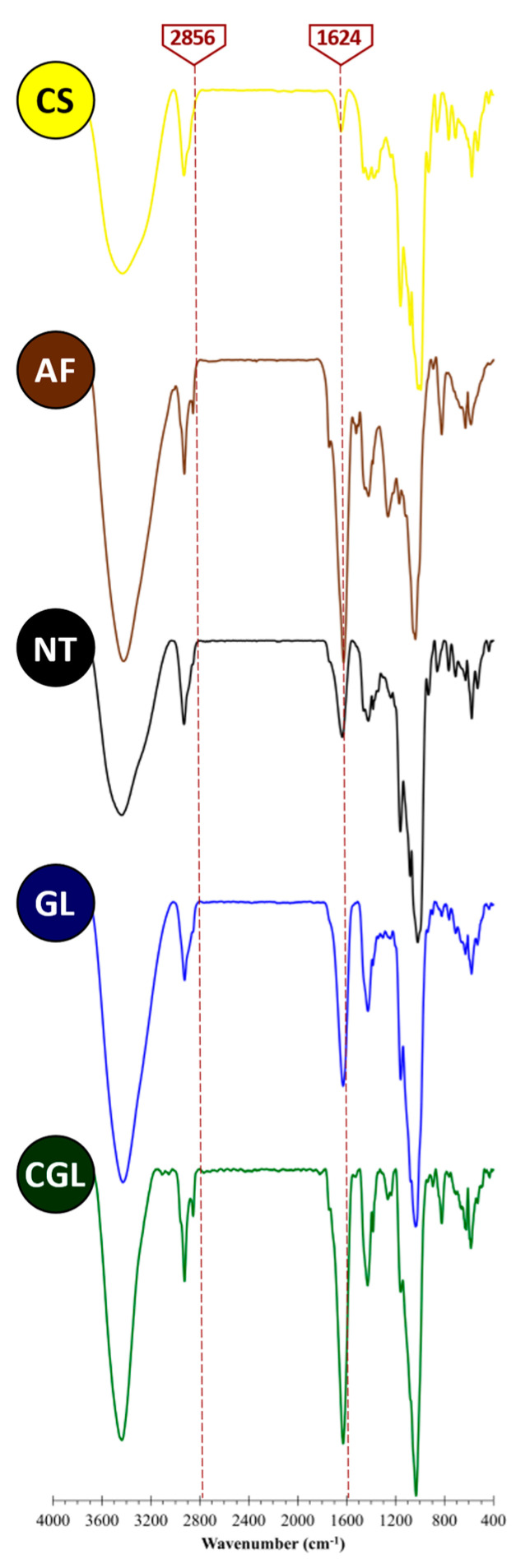
FT-IR spectra of control (CS, 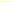 and AF, 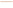) and AF–CS (1:1 ratio) samples (NT, 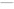, GL, 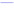, and CGL, 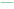 methods).

All samples showed a notorious peak at 3400 cm^–1^ that corresponded with the vibrational stretching of inter- and intra-molecular hydroxyl groups (–OH), Figure 5. However, around 2800 cm^–1^, AF and CGL showed two peaks not observed in other samples corresponding to hydrogen bridge bonds. Indeed, intensity values of this region are often used as a criterion to measure the amount (concentration) of hydrogen bridge bonds [34]. An explanation for the presence of these signals is that these bonds mainly are between polyphenol molecules forming complexes and the existence in CGL samples corroborated that polyphenols are entrapped in the gel structure, and these hydrogen bridge bonds remained intact.

The region around 1600 cm^–1^ corresponds to the characteristic peak of aromatic rings (stretching vibrations of C=C bonds). In this region, significantly (*p* < 0.05) different quantitative signals among samples with and without AF were found and were straightly related to polyphenols (phlorotannins) content (AF > CGL > GL > NT > CS samples). Particularly, CGL’s high peak intensity indicated the increasing retention by entrapment of polyphenols by CS, even after gelatinization treatment [35]. NT and GL samples, according to the literature [19,29,33], interact between AF and CS based on physical bonds. Physisorption has a relatively low degree of specificity and is therefore regarded as non-specific [10,36]. No new peaks were observed in the FT-IR spectra in AF–CS samples, demonstrating that interactions consisted of non-specific physical bonds.

### 3.3. Scanning Electron Microscopy (SEM)

**Figure 6:**
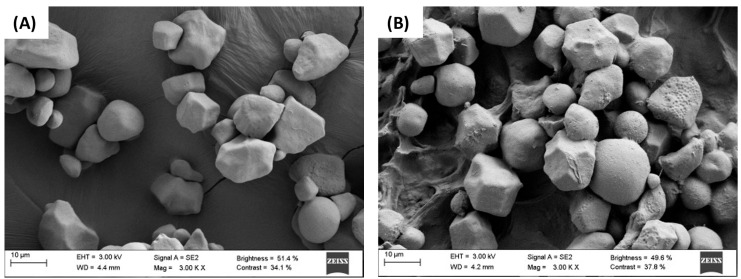

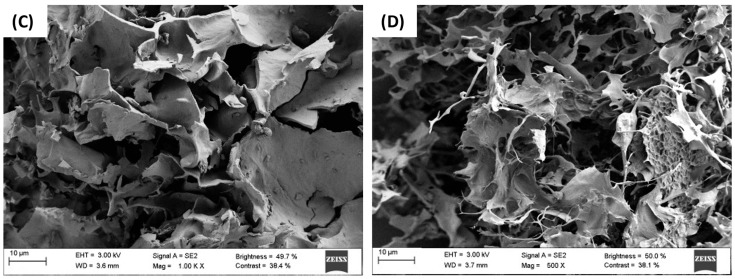
Cross-sectional morphologies of solid phase from CS (**A**) and AF–CS (1:1 ratio) samples (NT (**B**), GL (**C**), and CGL (**D**)).

SEM micrographs of CS and AF–CS blends (1:1 ratio) were carried out to observe the proposed physical bonding (NT, GL, and CGL) and trapping (CGL) interactions between AF and CS (Figure 6). CS control sample (Figure 6A) showed characteristic granular structures of native starch. In Figure 6B, the NT method showed starch granules and a heterogeneous and rough film that partially covered some particles and acted as a cohesive structure, forming bridges between granules. Both GL (Figure 6C) and CGL (Figure 6D) showed widespread and characteristic honeycomb gelled starch structures [10,32]. It was noted that gelled micrographs (GL and CGL) did not show any residual starch granules; therefore, full gelatinization was achieved. In fact, GL walls (Figure 6C) were thicker than those in CGL (Figure 6D), but the size of the holes did not show significant differences.

Additionally, micrographs of AF–CS blends showed some particles along granule surface and gel walls that were associated with *A. nodosum* particles. NT (Figure 6B) and GL (Figure 6C) showed these particles on the CS surface; meanwhile, CGL (Figure 6D) showed these particles below the wall surface, causing notorious prominent and dispersed bulges. These differences confirmed theories proposed where gelling of CS in the presence of AF (CGL method) entrapped AF particles and bioactive molecules inside the starch gel structure. The CGL method seems a suitable method to improve the bioactivity of starchy materials concerning NT or GL methods that were adsorbed on CS surfaces.

## 4. Conclusions

The production of functional foods employing edible seaweeds in rich starchy foods could have health benefits and preservation advantages. Composition and phase transitions promoted by heating operations, such as gelatinization, modify the phytochemical features, interactions, and cross-sectional morphologies of these bioactive starchy-based meals.

Starch showed different polyphenols retention features and yields depending on native or gelled structures. Results derived from FT-IR spectra and SEM micrographs show that physisorption of the polyphenols on starch is the main mechanism when seaweed flour is put in contact with native and gelled starch and adsorbed amount depends on the available surface area. Retention of polyphenols notoriously increases when starch gelatinization is carried out in the presence of seaweed flour because polyphenols are physically adsorbed on the surface of the starch gel and, additionally, they are trapped inside starch gel walls. The procedures used serve to quantify in a simple way the retention mode of bioactive molecules in gelled starch foods. Evaluation of antioxidant activities by DPPH, ABTS, and FRAP methods confirmed the reduction in bioactivity in the liquid phase by the retention of polyphenols by non-solubilized starch. These results provide insight into polyphenols–starch interactions to produce tunable starchy food materials. Additional research on these new bioactive products considering the different nature of polyphenols interactions (physisorption and entrapment) with starch could be necessary to understand better their antioxidant, anti-diabetic, and bioactive behavior during digestion.

## Figures and Tables

**Table 1:** Polyphenol sorption yields (Y_P_) ratios from NT, GL and CGL assayed methods.

AF–CS Ratio	GL/NT	CGL/NT	CGL/GL
1:0.5	1.75 ± 0.07 ^d^	2.70 ± 0.05 ^f^	1.54 ± 0.08 ^c^
1:1	1.45 ± 0.06 ^b,c^	1.99 ± 0.04 ^e^	1.37 ± 0.05 ^b,c^
1:2	1.34 ± 0.06 ^b,c^	1.84 ± 0.04 ^d,e^	1.28 ± 0.07 ^b^
1:25	1.02 ± 0.04 ^a^	1.02 ± 0.02 ^a^	1.00 ± 0.03 ^a^

NT method: AF (seaweeds flour) and native corn starch (CS) blending; GL method: AF and gelled CS blending; CGL method: CS gelatinized in the presence of AF. Different letters in columns indicate significant (*p* < 0.05) differences among samples.

**Table 2:** Parameters of the Halsey equation (Equation (5)).

Method	A	B	*R^2^*	RMSE
NT	6.0 ± 0.6	0.90 ± 0.08	0.98	3.57
GL	5.2 ± 0.7	0.53 ± 0.02	0.99	0.43
CGL	6.1 ± 0.4	0.33 ± 0.04	0.99	1.22

## Data Availability

The data presented in this study are available on request from the corresponding author.
